# A functional variant in the OAS1 gene is associated with Sjögren’s syndrome complicated with HBV infection

**DOI:** 10.1038/s41598-017-17931-9

**Published:** 2017-12-14

**Authors:** Xianjun Liu, Hongcun Xing, Wenjing Gao, Di Yu, Yuming Zhao, Xiaoju Shi, Kun Zhang, Pingya Li, Jiaao Yu, Wei Xu, Hongli Shan, Kaiyu Zhang, Wanguo Bao, Xueqi Fu, Sirui Yang, Shaofeng Wang

**Affiliations:** 1 The Bethune Institute of Epigenetic Medicine, The First Hospital of Jilin University, Changchun, China; 2 College of Life Sciences, The University of Jilin, Changchun, China; 3 Department of Hepatobiliary and Pancreatic Surgery, The First Hospital of Jilin University, Changchun, China; 4 The Research Center, The Second Hospital of Jilin University, Changchun, China; 5 The College of Pharmacy, The University of Jilin, Changchun, China; 6 Department of Burn Surgery, The First Hospital of Jilin University, Changchun, China; 7 Department of Clinical Laboratory, The First Hospital of Jilin University, Changchun, China; 8 Department of infectious Diseases, The First Hospital of Jilin University, Changchun, China; 9 Center of Pediatrics, Institute of Pediatrics, The First Hospital of Jilin University, Changchun, China

## Abstract

Hepatitis B virus (HBV) has been suspected to contribute to several autoimmune diseases, including Sjögren’s syndrome (SS), although the exact mechanism is unknown. The 2′–5′ oligoadenylate synthetase (OAS1) is one of the most important components of the immune system and has significant antiviral functions. We studied a polymorphism rs10774671 of OAS1 gene in Han Chinese descent. The minor allele G was significantly associated with a decreased risk for SS, anti-SSA-positive SS, and anti-SSA-positive SS complicated with HBV infection, which have not been seen in anti-SSA-negative SS and HBcAb-negative SS patients. Gene expression analysis showed that the risk-conferring A allele was correlated with lower expression of p46 and increased expression of p42, p48, and p44. A functional study of enzymatic activities revealed that the p42, p44, and p48 isoforms display a reduced capacity to inhibit HBV replication in HepG2 cells compared to the normal p46 isoform. Our data demonstrated that the functional variant, rs10774671, is associated with HBV infection and anti-SSA antibody-positive SS. The SAS variant switches the primary p46 isoform to three alternatives with decreased capacities to inhibit HBV replication. These data indicated that individuals harboring the risk allele might be susceptible to hepatitis B infection and SS development.

## Introduction

Sjögren’s syndrome (SS) is a common, systemic autoimmune disease, with a prevalence of approximately 0.5% in the general population^1,2^. The clinical features of the disease include lymphocytic infiltration of exocrine glands (mainly salivary and lacrimal), the activation of B-lymphocytes, and the production of anti-SSA and anti-SSB autoantibodies^3^. Loss of secretory activity of salivary and lacrimal glands has been observed, resulting in dry mouth and eyes. Other systems or organs, such as the musculoskeletal and nervous systems, liver, skin, and kidneys, can also be affected. The causative mechanisms of SS are not yet fully understood. Accumulating evidence has shown that both genetic and environmental factors contribute to SS development^4^.

Recently, the dysregulation of interferon (IFN) signaling pathways has been observed in the salivary glands and peripheral blood of SS patients^5–8^. IFNs are key immune mediators involved in viral defense and immune response activation^9–11^. Additionally, recent genome-wide association studies and candidate gene scans have indicated the importance of multiple genetic loci in SS pathogenesis, in which numerous IFN-regulated genes have shown significant associations with SS^12^.

*OAS1*, induced by IFN, encodes the key antiviral enzyme 2′–5′ oligoadenylate synthetase 1^13^. OAS1 is a well-known molecule that restricts viral infection by degrading viral RNA in combination with RNase L, resulting in the inhibition of viral replication^14,15^. Four OAS1 isoforms have been identified^16^. A single-nucleotide polymorphism (SNP), rs10774671, at the intron 5 splice acceptor site (SAS) of the *OAS1* gene affects the production of various OAS1 isoforms^17^. The G reference allele produces *OAS1* transcript variant 1 (TV1), which results in the production of the OAS1 p46 isoform; the alternate A allele changes the SAS site, resulting in the loss of the canonical SAS, and produces three transcript variants, TV2 (p42), TV3 (p48), and TV4 (p44), which encode different isoforms with altered enzymatic activities^14,16,17^.

The SAS variant, rs10774671, has been associated with multiple autoimmune diseases, including type 1 diabetes (T1D)^18–20^ and multiple sclerosis (MS)^21,22^. A recent large-scale association study was just published and showed a significant association of variant at the *OAS1* locus in SS in European population^23,24^. By comparing 835 T1D and 401 healthy siblings (subjects were collected from 574 families of Danish, Canadian, and American heritage), the G allele of rs10774671 was found to significantly increase the risk of developing T1D^18^. In MS, contradictory results have been reported. A Spanish study showed that the G allele is associated with increased risk for MS^21^; however, an Irish study showed that the G allele plays a protective role^25^. These data suggested that rs10774671 has distinct effects on various autoimmune diseases and ancestries^26^. To date, there is only limited information on the nature of the SAS variant of the *OAS1* gene and susceptibility to SS^23^. Thus, a population-based case-control study to evaluate the association of SS and the *OAS1* gene is critically needed.

Hepatitis B virus (HBV) infection is highly prevalent in human populations. Of the 350 million individuals worldwide infected with HBV, 33% reside in China^27^. Although there is accumulating evidence indicating that hepatitis C virus (HCV) infection contributes to the etiology of SS^28,29^, there is a limited number of reports regarding the nature of the association between SS and HBV infection^30–32^. Two large cohort studies have suggested that HBV infection might be more prevalent in patients with SS than in the general population^30,31^. However, the pathogenic role of HBV in SS-susceptible individuals has not been well characterized. Between 2005 and 2016, we established a cohort of 588 patients with SS and 1455 healthy controls to study genetic factors that influence the risk for SS. To evaluate the pathogenic role of HBV in triggering SS in susceptible individuals, we analyzed the clinical information for 368 anti-SSA-positive SS patients for HBV infection by screening their anti-HBc antibody status. Of the 68 tested SS patients, 30 SS patients were previously infected by HBV (HBcAb+), and 38 SS patients were negative for HBcAb. Therefore, we set out to evaluate the genetic association of the SAS variant of the *OAS1* gene in a case-control study complicated by HBV infection.

## Results

### The rs10774671 SNP is associated with SS and anti-SSA-positive SS

The demographics of the 588 cases and 1455 independent controls enrolled in this study are shown in Supplementary Table 1. There were no significant differences between the case and control subjects in terms of mean age or gender distribution. All cases and controls in the analysis are self-reported Han Chinese. As shown in Table 1, single-marker association was performed using a logistic regression with multiple models. We observed an association of variant rs10774671 in SS (*P* = 0.039 for an additive model, *P* = 0.01 for a dominant model, and *P* = 2.9 × 10^−4^ for a recessive model), with an effect size of odds ratio (OR) = 0.85. Given that data reflecting the anti-SSA autoantibodies of 429 SS patients were available, we further stratified SS patients based on their anti-SSA autoantibodies status. We observed a significant association of rs10774671 with anti-SSA-positive SS (*P* = 8.0 × 10^−5^) with an effect size of OR = 0.70. However, the association was not seen in anti-SSA-negative SS (*P* = 0.1725) (Table 1). These results demonstrated, for the first time, the association of *OAS1* in SS patients and anti-SSA-positive SS patients in Han Chinese. The G allele of rs10774671 plays a protective role, and the A allele confers risk for SS. In our cohort, the frequencies of the risk allele A were 0.71 in controls and 0.78 in SS cases. A similar pattern of association was observed in SS patients with European ancestry, in which the frequencies of the risk allele A were 0.65 in controls and 0.72 in SS cases^23,24^. These findings suggest that this genetic risk factor could influence risk of SS in both European and Asian populations.

**Table 1:** The genetic association of rs10774671 in SS and anti-SSA positive SS.

	N	MAF	Genotype			SS cases vs Controls OR (95% CI) *P*-value	Anti-SSA + SS vs Controls OR (95% CI) *P*-value	Anti-SSA − SS vs Controls OR (95% CI) *P*-value	Anti-SSA + SS vs Anti-SSA – SS OR (95% CI) *P*-value
			GG	GA	AA				
Controls	1455	0.2948	140	578	737				
SS cases	588	0.2628	28	253	307	0.85 (0.73–0.99)	0.70 (0.58–0.85)	1.30 (0.88–1.88)	0.62 (0.42–0.91)
Anti-SSA + SS	368	0.2255	12	142	214	**2**.**9 × 10**^**−4**^	**8**.**0 × 10**^**−5**^	0.1725	**0**.**0025**
Anti-SSA − SS	61	0.3525	4	35	22				

Note: MAF: minor allele frequency; OR: odds ratio; CI: confidence interval.

### The variant rs10774671 is associated with anti-SSA-positive SS complicated by HBV infection with a reduced effect size

To evaluate the role of rs10774671 in the pathogenesis of anti-SSA-positive SS complicated with HBV infection, we classified the anti-SSA-positive SS patients into two groups: anti-SSA+/HBcAb+ (n = 30) and anti-SSA+/HBcAb− (n = 38). Comparisons were made between anti-SSA+/HBcAb+ SS, anti-SSA+/HBcAb− SS, and controls. We performed an association study using Fisher’s exact test to account for small sample sizes. As shown in Table 2, the protective allele G of the rs10774671 SNP was significantly associated with anti-SSA+/HBcAb+ SS (*P* = 0.0059), with an effect size of OR = 0.36. Interestingly, this association was not seen in the anti-SSA+/HBcAb− SS samples (*P* = 0.3746, OR = 1.24), although the sample size of the anti-SSA+/HBcAb– SS group (n = 38) was greater than the anti-SSA+/HBcAb + SS group (n = 30). Our data showed that the protective allele G was less common in the anti-SSA+/HBcAb + SS group (frequency of the G allele = 0.13), and the altered risk allele A was strongly enriched in the anti-SSA+/HBcAb + SS group (frequency of the A allele = 0.87). These results demonstrated that the *OAS1* rs10774671 A allele conferred risk for SS patients with HBV infection compared to SS patients having no HBV infection. In contrast to the effect sizes of the association signals of rs10774671 in SS patients (OR = 0.85) and in anti-SSA-positive SS (OR = 0.70), the effect size in HBV-infected SS patients was decreased (OR = 0.36). These data suggested that the A allele of genetic variant rs10774671 contributes to the risk for SS. Therefore, HBV infection might play a pathogenic role in genetically predisposed individuals at the *OAS1* locus to develop SS.

**Table 2:** The association of rs10774671 in SS complicated by HBV.

	N	MAF	Genotype			HBc + SS vs controls OR (95% CI) *P*-value	HBc − SS vs controls OR (95% CI) *P*-value	HBc − SS vs HBc + SS OR (95% CI) *P*-value
			GG	GA	AA			
Controls	1455	0.29	140	578	737			
SS cases	588	0.26	28	253	307	0.36 (0.18–0.79)	1.24 (0.63–2.38)	0.29 (0.06–0.70)
SSA + HBc + SS	30	0.13	0	8	22	**0**.**0059**	0.3746	**0**.**0055**
SSA + HBc − SS	38	0.34	2	22	14			

Note: MAF: minor allele frequency; OR: odds ratio; CI: confidence interval; HBc: hepatitis B core protein.

### OAS1 gene expression is significantly increased in anti-SSA-positive patients

**Figure 1:**
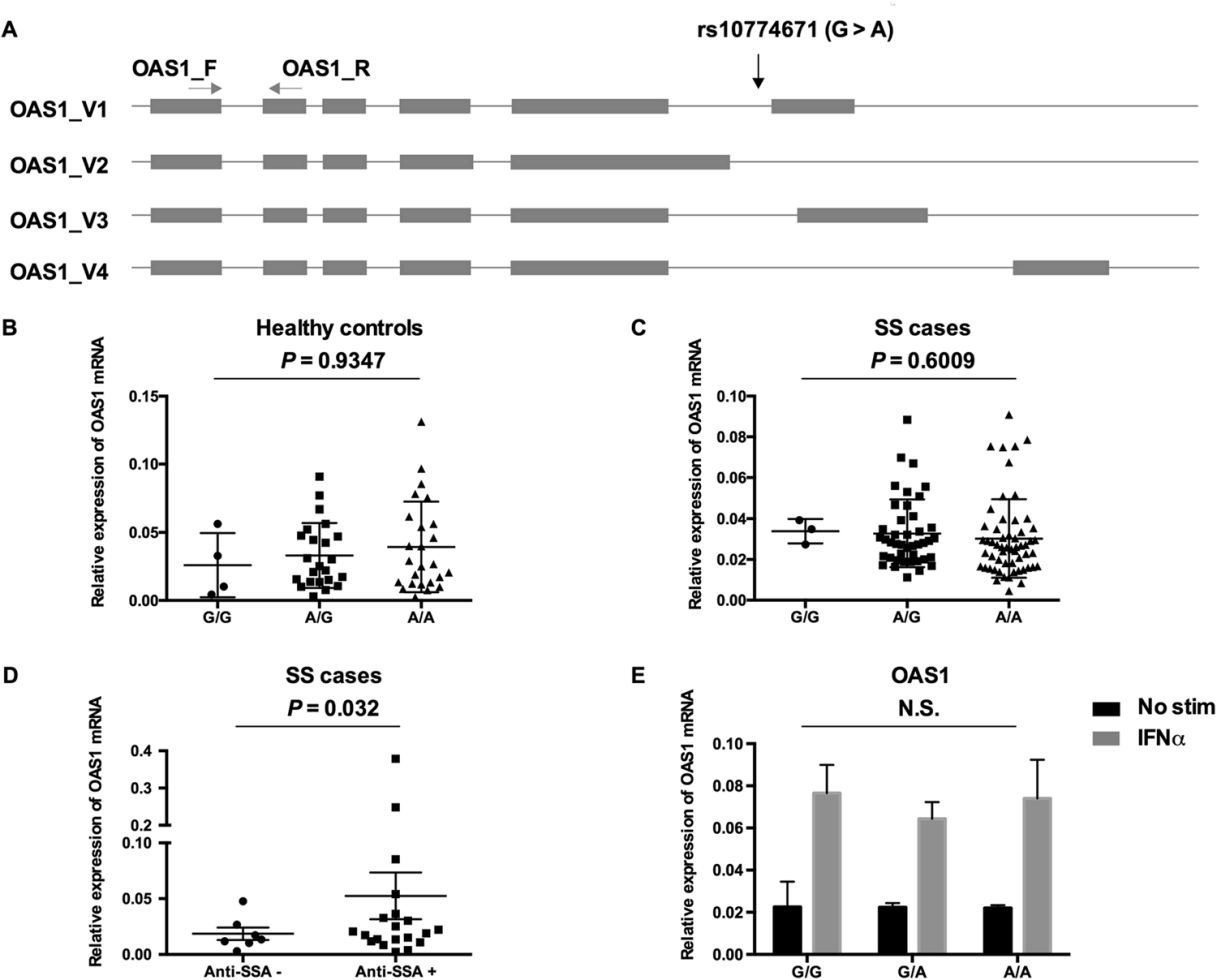
Gene expression analysis of *OAS1* in PBMC. (**A**) The schismatic figure shows the relative location of rs10774671 and *OAS1* gene. Pairs of primers, OAS1_F and OAS1_R for detecting total expression of *OAS1* were indicated. (**B**,**C**) The effect of SS-associated rs10774671 on *OAS1* mRNA expression in healthy controls and SS cases was determined by RT-qPCR, respectively. Each data point represents an individual subject, and the bars show the standard deviation (SD). *P* values were calculated by using one-way ANOVA. (**D**) The comparison of *OAS1* expression in PBMC between anti-SSA positive and anti-SSA negative SS cases. (**E**) 12 EBV-transformed B cell lines with various genotypes at 10774671 were stimulated by IFNα. Statistically differences between three groups were calculated by one-way ANOVA. *P* value < 0.05 was considered significant.

Our data demonstrated a significant association between the variant rs10774671 and SS in Han Chinese. To begin to evaluate the role of rs10774671 in SS pathogenesis, a bioinformatic analysis using Haploreg (4.1)^33,34^ was performed to identify variants that are in linkage disequilibrium (LD) with rs10774671. As shown in Supplementary Table 2 and Supplementary Figure 2, 98 variants in Asian individuals spanning the *OAS1*-*OAS3* locus were correlated with rs10774671, with an *r*^2^ ≥ 0.99. These data suggested that an rs10774671-tagged haplotype, spanning the *OAS1*-*OAS3* locus carrying 99 genetic variants, is associated with SS. Therefore, we evaluated the role of rs10774671 in regulating gene expression of *OAS1* and *OAS3* in peripheral blood mononuclear cells (PBMCs) from patients with SS (n = 54) and from healthy controls (n = 104) by RT-qPCR assays according to the methods in our previous studies^35–38^. In healthy individuals and SS cases, the genotypes of rs10774671 do not influence total expression of *OAS1* and *OAS3* (Fig. 1B and C and Supplementary Figure 3). Additionally, we generated 12 Epstein-Barr virus (EBV)-transformed B cell lines using PBMCs from patients with SS and compared gene expression levels of *OAS1* and *OAS3* stimulated with IFNα for 2 hours. Messenger RNA expression levels of *OAS1* and *OAS3* were compared between GG (n = 4), GA (n = 4), and AA (n = 4) groups. There were no significant differences of *OAS1* (Fig. 1E) and *OAS3* (Supplementary Figure 4) gene expression between groups. However, when samples were classified by anti-SSA autoantibodies status, individuals who tested positive for anti-SSA antibodies showed a significant increase in total *OAS1* gene expression in PBMCs (*P* = 0.032) (Fig. 1D).

### The major allele A of rs10774671 leads to an increased risk for SS by regulating pre-mRNA splicing of the OAS1 gene

**Figure 2:**
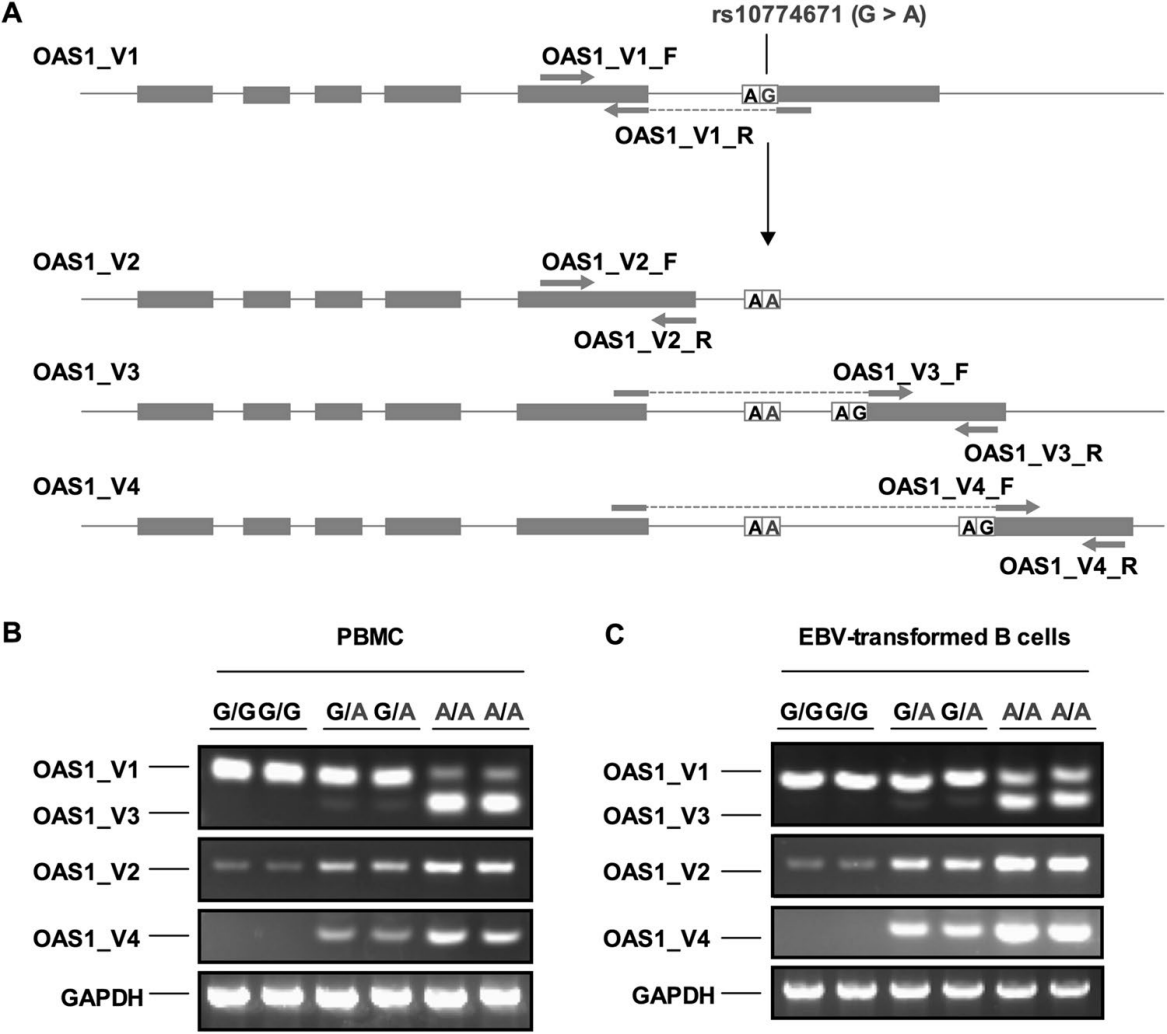
The expression of isoforms of OAS1 in PBMC and EBV-transformed B cell lines from patients with SS. (**A**) The schismatic figures show the four transcript variants of OAS1 mRNA. The grey boxes represent exons of *OAS1*. The SAS SNP rs10774671 determines the splicing of *OAS1* mRNA. Four pairs of primers designed specifically detect each isoform of OAS1 are indicated. (**B**) Quantitative PCR analyses of expressions of isoforms in PBMC from 6 SS patients and in 6 EBV-transformed B cell lines (**C**) were performed. The isoforms (OAS1_V1 and OAS1_V3) were amplified with a same pair of primers and were resolved on the same gel. The isoforms OAS1_V2 and OAS1_V4 were amplified with two pairs of primers designed specifically for the V2 and V4 and were resolved separately. The GAPDH amplicon from each sample was used as a loading control.

Given that the risk-conferring A allele of rs10774671 is a splice acceptor site variant located at the junction between intron 5 and exon 6 and may switch the primary normal isoform to various alternatives, we assessed whether the altered allele A influences the expression of various isoforms of OAS1 in PBMCs and EBV-transformed B cell lines from patients with SS. Primers that specifically detect different transcript variants (TVs) of *OAS1* were designed and are listed in Supplementary Table 3. Quantitative PCR analyses were performed to detect expression of specific TVs of *OAS1*. As shown in Fig. 2, the switching of reference allele G to risk-conferring allele A results in reduced expression of the TV1 (p46) isoform and increased expression levels of TV2, TV3, and TV4. To further confirm this finding, we replicated the gene expression analyses in PBMCs obtained from 20 patients with SS. As shown in Supplementary Figure 5, we consistently observed that the risk-conferring allele A reduces expression of the TV1 (p46) isoform and increases the expression levels of the three alternatives.

### Risk allele A of the rs10774671 SNP demonstrated reduced activity in the clearance of HBV infection

**Figure 3:**
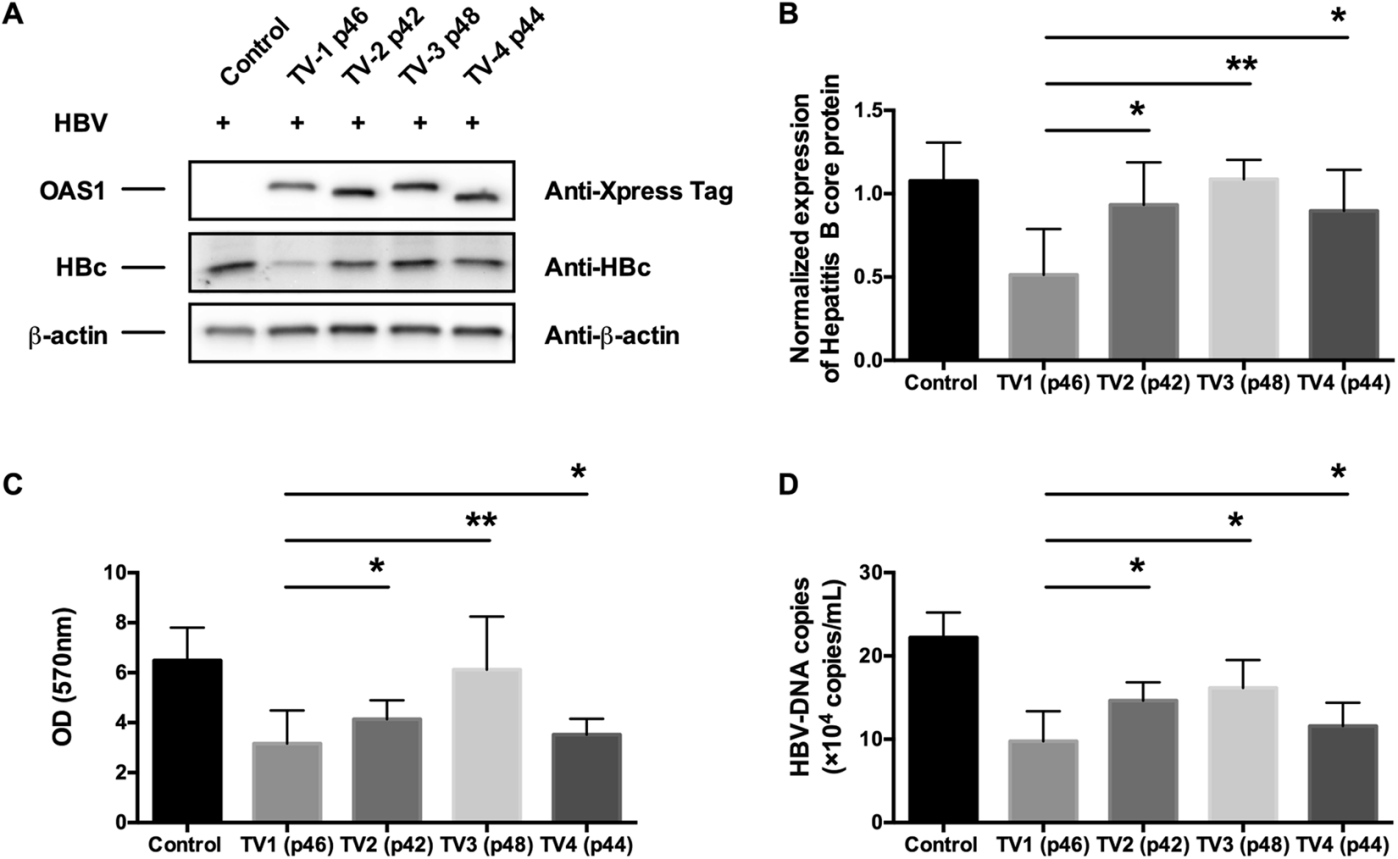
Functional analyses of the anti-virus activities of various isoforms of OAS1 in HepG2 cells. (**A**) The OAS1 proteins, HBV core protein, and actin were measured by western blot analysis. (**B**) Optical densities of the hepatitis core protein were analyzed using Image-J software. The data are the mean ± SD (n = 3). (**C**) Hepatitis B surface antigen (HBsAg) in the culture supernatant was analyzed by enzyme-linked immunosorbent assay (ELISA). (**D**) HBV-DNA in the culture supernatant was measured by RT-qPCR. Data represent the mean ± SD of three experiments. Comparisons were made between TV2, TV3, and TV4 versus TV1. Statistically differences were calculated by student t-test. Asterisks *indicates *P* < 0.05 and double asterisks **indicates *P* < 0.01.

Previous studies on enzymatic activities of various isoforms of the *OAS1* gene product in clearance of hepatitis C virus^39,40^ and West Nile virus^41^ showed that the p46 isoform is significantly more active than the TV3 (p48) isoform. Additionally, it is known that *OAS1* plays a critical role in restricting HBV infection and replication^42^. In this study, we observed that the rs10774671 SNP is significantly associated with SS complicated with HBV infection. Therefore, we hypothesized that the risk-conferring allele A, which is associated with reduced OAS1 activity, is inefficient in the clearance of HBV in SS-susceptible individuals and influences the risk for SS with HBV infection. To test this hypothesis, we co-transfected HepG2 cells with an HBV-producing plasmid and *OAS1* isoform-expressing vectors. Six days post-transfection, we determined the expression levels of the OAS1 isoforms and the hepatitis B core protein in HepG2 cells by western blotting and measured the HBV-DNA by RT-qPCR. As shown in Fig. 3, the various isoforms of *OAS1* were expressed evenly; however, we observed that the TV2, TV3, and TV4 isoforms showed significantly lower capacity to restrict HBc protein expression in cells than TV1 (p46) (Fig. 3). To evaluate the activities of *OAS1* in restricting HBV surface antigen release in HepG2 cells, we measured the levels of HBsAg in the culture medium by ELISA. As shown in Fig. 3C, the levels of HBsAg in the culture media in TV2-, TV3-, and TV4-expressing cells were significantly higher than those in the media in TV1-expressing cells. Our data demonstrated that the A allele-associated isoforms produce significantly lower activity to inhibit HBV replication than TV1 (p46). These data indicated that individuals harboring the risk allele might be susceptible to hepatitis B infection and SS development.

## Discussion

**Figure 4:**
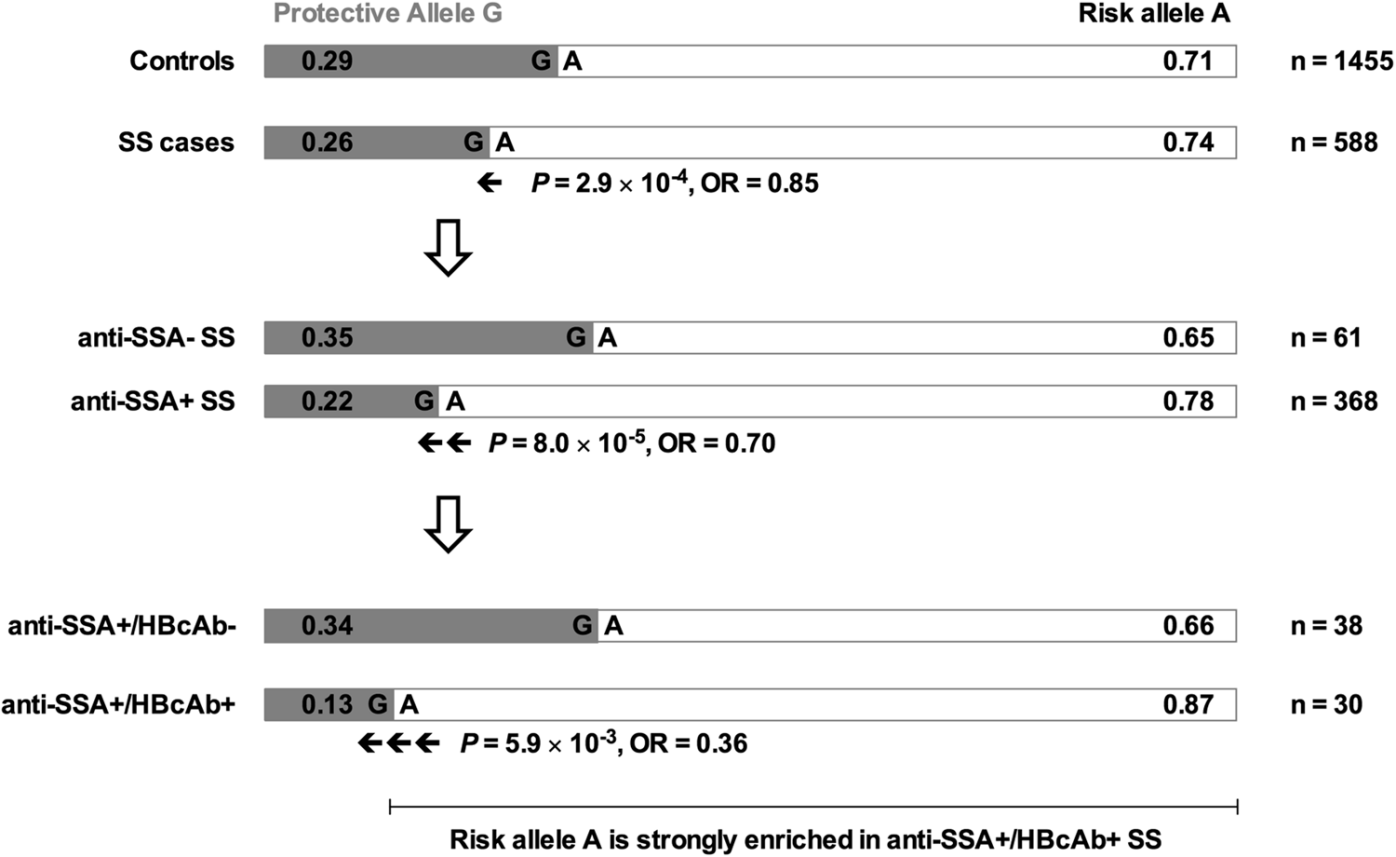
The risk allele A or rs10774671 is strongly enriched in the anti-SSA+/HBcAb + SS patients. The grey boxes represent the protective allele G and the white boxes represent the risk allele A. The allele frequencies of each allele are indicated in the boxes. P value for each analysis was based on the comparison of indicated group with healthy controls.

Little is known about the etiology and pathogenesis of SS, partially due to the complexity and heterogeneity of the disease mechanisms^1,29^. Dysregulation of IFN signaling pathways has been observed in patients with SS and makes the IFN-inducible gene *OAS1* a good candidate risk gene for SS^9,12^. Recently, a large-scale genetic association study was just published and demonstrated a significant association of variant at the *OAS1* gene with SS in European population^24^. In addition to the genetic associations of *OAS1* with T1D^18–20^, MS^21,22^, and SS in European, we demonstrated a genetic association of rs10774671 of the *OAS1* gene with SS (OR = 0.85) in Chinese Han descent, especially with anti-SSA autoantibody-positive SS (OR = 0.70). Among SS patients who tested positive for the hepatitis B core protein antibody, the effect size of the association was reduced to OR = 0.36 (Fig. 4). The risk allele A is strongly enriched in anti-SSA+/HBcAb-positive SS patients. Interestingly, the genetic association was not seen in anti-SSA+/HBcAb - negative SS patients. Further functional studies revealed that the risk A allele leads to the changing of a consensus sequence of the splicing accepter site at intron 5 of *OAS1*, resulting in the production of three alternative isoforms with a reduced capacity to inhibit HBV replication in HepG2 cells. The data from these functional studies are consistent with our findings in the genetic association study.

The association of SS with HCV has resulted in an intense debate over the past decade. In 1992, researchers found the first histological evidence of SS in 16 of 28 patients with chronic HCV infection^43^. Since then, numerous studies have shown significant associations of SS with HCV. However, the association of SS and HBV has not been established, although both viruses are highly prevalent. Interestingly, there are a number of case reports demonstrating that SS occurs after hepatitis B vaccination and suggesting a role of HBV in SS pathogenesis^44^. A recent study on the prevalence of HBV infection in patients with SS (603 cases) in Spain showed a slight increase in the percentage of HBV infection in SS patients (HBsAg + 0.83%) compared to the population controls (HBsAg + 0.7%)^31^. Additionally, a study of SS patients from Taiwan (9,629 cases) infected with HBV showed that the HBV infection was more frequent in SS patients (4.3%) than in the healthy population (3.6%, 38,516 controls)^30^. Our data indicated that there is an increased incidence of HBV in SS patients carrying the A allele. In addition to the HBV, viruses like HIV, HTLV-1, and HDV, are also shown to influence risk for SS. However, the patients in our cohort are HIV and HDV negative. The serological data in our cohort is not available for the HTLV-1, therefore, infections of other SS-associated viruses in patients should be considered as they might correlate with the risk variant of the *OAS1* gene.

The SNP rs10774671 at intron 5 of the *OAS1* gene affects pre-mRNA splicing and the enzymatic activities of various isoforms of OAS1, which have been intensively studied in the context of viral infections, including HCV. However, the expression profiles of OAS1 isoforms in SS patients and the activities of different OAS1 isoforms in controlling HBV replication remain to be elucidated. Our findings in the current study revealed a molecular mechanism by which rs10774671 impacts the pre-mRNA splicing of *OAS1* in patients with SS, which was similar to previous findings in other tissues and diseases^17,19^. Failure to clear virus might lead to a chronic infection that drives the sustained overexpression of IFNs, which is associated with increased risk of SS^24,45^. On the other hand, viral proteins may also indirectly cause IFN production through adaptive immune responses^45,46^. Further mechanistic study of hepatitis B viral proteins may or may not directly influence salivary gland function *in vitro*, and animal models are fundamental to understand the effect link between HBV and development of Sjögren’s syndrome.

In summary, we demonstrated a genetic association of SNP rs10774671 in the *OAS1* gene in SS, anti-SSA-positive SS, and HBV-positive SS patients, with an enhanced effect size. A functional study of the risk variant demonstrated that the SS-associated risk allele A leads to decreased expression of the normal isoform of OAS1 (TV1, p46) and results in the expression of alternative isoforms with reduced activities in inhibiting HBV replication in HepG2 cells. These findings provide significant insight into the mechanistic understanding of how risk variant might result in a reduced ability to the clearance of HBV, leading to a chronic infection and constitutive activation of the IFN signaling, influence risk for SS in both Asian and European populations.

## Materials and Methods

### Patients and samples

The study population consisted of 2043 adult unrelated Chinese, including 588 SS cases and 1455 matched population controls (all participants in this study self-reported to be Han Chinese). Individuals were recruited between March 2005 and August 2016 from The First Hospital of Jilin University in China. All SS patients were diagnosed according to the standards defined by criteria of the American European Consensus Group in 2002 and were enrolled in this study (Table 1). There was no sex and age restriction (Supplementary Table 1). Four hundred twenty-nine patients were tested for anti-SSA and anti-SSB autoantibodies, including 368 (62.6%) with anti-SSA antibodies, 182 (30.9%) with anti-SSB antibodies, 179 (30.4%) with both anti-SSA and anti-SSB antibodies, and 311 (52.9%) with previous or current systemic complications. The ethics committee of The First Hospital of Jilin University approved this protocol. Written informed consent was obtained from all study participants.

#### Patients with SS complicated with infections of hepatitis viruses

Viruses including HIV, HTLV-1, HCV, and HDV have been associated with increased risk for SS. We checked available serological data in our cohort and found that seventy-six SS patients were tested for HCV infection, and only 1 patient was positive. All subjects including cases and controls are negative for HIV and HDV. Tests for HIV and HDV antibody detection are accomplished by Abbott HIVAB HIV-1/2 (rDNA) EIA kit and Cusabio HDV IgG ELISA kit respectively as instruction’s suggestive protocols (Abbott, Tokyo, Japan; Cusabio, Guangzhou, China). For HTLV-1, there is no serological information available. Of the 368 anti-SSA antibody-positive SS cases, 68 patients were given the serological tests for HBsAb and HBcAb. Given that the HBV vaccination is routinely administered to people in China, a positive HBsAb test could not distinguish infection or vaccination of HBV. Therefore, we classified 68 patients with serological test results into two groups based on their anti-HBc status. Patients who were positive for anti-HBc demonstrated exposure to HBV. Because anti-HBc can persist for life, these patients either had an active hepatitis B infection or have had hepatitis B in the past. Additionally, the patients with anti-HBc antibodies also tested for the HBV core proteins. All patients were negative for the HBV core protein suggesting there is no active infection. As shown in Table 2, 30 anti-SSA-positive SS patients were positive for HBcAb, and 38 anti-SSA-positive SS patients were negative for HBcAb.

### SNP genotyping

Genomic DNA was extracted from PBMCs using a DNA-Beads-400 DNA extraction kit (Zhiang Biotech, Changchun, China), according to the manufacturer’s instructions. The PCR primers were designed as listed in Supplementary Table 3. Genomic DNA from each sample was amplified, and the genotype at rs10774671 for each sample was determined using a TaqMan SNP genotyping assay (Thermo Fisher Scientific Inc. Beijing, China) on an Applied Biosystems™ OpenArray™ real-time PCR instrument. In addition to the TaqMan SNP genotyping assays, several PCR products were randomly selected and subjected to Sanger sequencing to confirm the results (Supplementary Figure 1).

### RNA isolation and quantitative RT-PCR

Total RNA from PBMCs was isolated using TRIzol (Invitrogen Inc., Carlsbad, CA, USA) according to the manufacturer’s instructions. The concentrations of total RNA were determined by NanoDrop, and samples were diluted with 10 ng/μL of MS2-RNA (Hoffmann-La Roche, Inc., Nutley NJ, USA) to a final concentration of 100 ng/μL. cDNA from each individual was synthesized using iScript cDNA Synthesis Kits (Bio-Rad Laboratories, Inc., Hercules, CA, USA). Quantitative RT-PCR was performed to determine the mRNA expression levels of four OAS1 isoforms. The human *GAPDH* gene was used in quantitative RT-PCR as a control.

### Statistical analysis

Single-marker associations were assessed using the logistic regression function in Plink, version 1.09. The Hardy-Weinberg proportion test *P* value of rs10774671 in the controls was greater than 0.01. For the associations between rs10774671 and SS and anti-SSA-positive SS, multiple models were used. The associations of rs10774671 in anti-SSA-positive SS complicated with HBV were performed using Fisher’s exact test, given that a small sample size was available for this particular analysis. ORs and 95% confidence intervals (CIs) were calculated to assess the relative risk conferred by a allele and genotype. The comparisons of the mRNA expression levels of *OAS1* and the different isoforms in PBMCs and EBV-transformed B cell lines between different genotypes were performed using a non-parametric Kruskal-Wallis test with correction for multiple comparisons. A *P* value less than 0.05 was considered statistically significant.

### Molecular cloning of *OAS1*

To amplify DNA segments encoding different isoforms of *OAS1*, we designed a group of primers for initial and nested PCRs. A human B cell cDNA library was used as the template, and PCR was performed with high-fidelity Phusion polymerase (Thermo Fisher Scientific Inc. Beijing, China). The PCR products were cloned into the pBluescript-KS vector. Plasmid DNAs were purified from *E*. *coli* clones using a DNA mini-prep kit (Thermo Fisher Scientific Inc. Beijing, China). The correct construct was selected based on restriction enzyme digestions. DNA sequencing analyses with T7 and T3 primers then verified the entire sequence of the DNA insert. DNA segments encoding four isoforms of OAS1 were then sub-cloned into the pCDNA3.1 expression vector for the follow-up functional studies.

### Anti-HBV activity assays

HepG2 cells were maintained in complete Dulbecco’s Modified Eagle’s medium (DMEM, Gibco-BRL, CA) containing 10% fetal bovine serum (FBS, Hyclone, Fisher, PA), 100 units/mL of penicillin, and 100 mg/mL of streptomycin in a humidified incubator with 5% CO_2_ at 37 °C. Co-transfection of HepG2 cells was performed with expression vectors encoding various isoforms of OAS1 along with the HBV-producing plasmid pCMVayw-HBV. The culture medium was replaced every three days. Six days after transfection, the cells were harvested for HBV core protein assays. The supernatants were harvested for detecting HBV antigens using ELISA, and DNA from the cells was isolated for determination of HBV-DNA presence by quantitative PCR. The expression levels of the different isoforms of OAS1 and the HBV core protein in HepG2 cells were determined using a standard western blot procedure using an antibody against the co-expressed tag with OAS1 and anti-HBc antibody. β-actin was used as a loading control for the experiments. β-actin expression was detected and was used as a loading control for each lane. Three independent experiments were performed to determine the statistically significant differences.

All methods were performed in accordance with the institutional and national guidelines, regulations, and approvals.

### Data availability

All data generated or analysed during this study are included in this published article (and its Supplementary Information files).

## Electronic supplementary material

Supplementary Dataset

## References

[1] Brito-Zerón P (2016). Sjögren syndrome. Nature Reviews Disease Primers.

[2] Ice JA (2012). Genetics of Sjögren’s syndrome in the genome-wide association era. Journal of autoimmunity.

[3] Joachims, M. L. *et al*. Single-cell analysis of glandular T cell receptors in Sjögren’s syndrome. *JCI Insight* **1**, 10.1172/jci.insight.85609. 10.1172/jci.insight.85609 PMC4922426 27358913

[4] Burbelo PD, Ambatipudi K, Alevizos I (2014). Genome-wide association studies in Sjögren’s Syndrome: what do the genes tell us about disease pathogenesis?. Autoimmunity reviews.

[5] Båve U (2005). *Activation of the type I* interferon system in primary Sjögren’s syndrome: A possible etiopathogenic mechanism. Arthritis & Rheumatism.

[6] Hjelmervik TOR, Petersen K, Jonassen I, Jonsson R, Bolstad AI (2005). Gene expression profiling of minor salivary glands clearly distinguishes primary Sjögren’s syndrome patients from healthy control subjects. Arthritis & Rheumatism.

[7] Emamian ES (2009). Peripheral blood gene expression profiling in Sjögren’s syndrome. Genes and immunity.

[8] Peck AB, Nguyen CQ (2012). Transcriptome Analysis of the Interferon-Signature Defining the Autoimmune Process of Sjögren’s Syndrome. Scandinavian journal of immunology.

[9] Li, H., Ice, J., Lessard, C. & Sivils, K. Interferons in Sjögren’s Syndrome: Genes, Mechanisms, and Effects. *Frontiers in Immunology* **4**, 10.3389/fimmu.2013.00290 (2013). 10.3389/fimmu.2013.00290 PMC3778845 24062752

[10] González-Navajas JM, Lee J, David M, Raz E (2012). Immunomodulatory functions of type I interferons. Nature reviews. Immunology.

[11] Samuel CE (2001). Antiviral Actions of Interferons. Clinical Microbiology Reviews.

[12] Lessard Christopher J, Li He, Adrianto Indra, Ice John A, Rasmussen Astrid, Grundahl Kiely M, Kelly Jennifer A, Dozmorov Mikhail G, Miceli-Richard Corinne, Bowman Simon, Lester Sue, Eriksson Per, Eloranta Maija-Leena, Brun Johan G, Gøransson Lasse G, Harboe Erna, Guthridge Joel M, Kaufman Kenneth M, Kvarnström Marika, Jazebi Helmi, Graham Deborah S Cunninghame, Grandits Martha E, Nazmul-Hossain Abu N M, Patel Ketan, Adler Adam J, Maier-Moore Jacen S, Farris A Darise, Brennan Michael T, Lessard James A, Chodosh James, Gopalakrishnan Rajaram, Hefner Kimberly S, Houston Glen D, Huang Andrew J W, Hughes Pamela J, Lewis David M, Radfar Lida, Rohrer Michael D, Stone Donald U, Wren Jonathan D, Vyse Timothy J, Gaffney Patrick M, James Judith A, Omdal Roald, Wahren-Herlenius Marie, Illei Gabor G, Witte Torsten, Jonsson Roland, Rischmueller Maureen, Rönnblom Lars, Nordmark Gunnel, Ng Wan-Fai, Mariette Xavier, Anaya Juan-Manuel, Rhodus Nelson L, Segal Barbara M, Scofield R Hal, Montgomery Courtney G, Harley John B, Sivils Kathy L (2013). Variants at multiple loci implicated in both innate and adaptive immune responses are associated with Sjögren's syndrome. Nature Genetics.

[13] Rios JJ (2007). Characterization of the equine 2′-5′ oligoadenylate synthetase 1 (OAS1) and ribonuclease L (RNASEL) innate immunity genes. BMC Genomics.

[14] Zhu J, Ghosh A, Sarkar SN (2015). OASL – a new player in controlling antiviral innate immunity. Current opinion in virology.

[15] Zhu J (2014). Antiviral activity of human oligoadenylate synthetases-like (OASL) is mediated by enhancing retinoic acid-inducible gene I (RIG-I) signaling. Immunity.

[16] Pulit-Penaloza JA, Scherbik SV, Brinton MA (2012). Activation of Oas1a gene expression by type I IFN requires both STAT1 and STAT2 while only STAT2 is required for Oas1b activation. Virology.

[17] Bonnevie-Nielsen V (2005). Variation in Antiviral 2′,5′-Oligoadenylate Synthetase (2′5′AS) Enzyme Activity Is Controlled by a Single-Nucleotide Polymorphism at a Splice-Acceptor Site in the OAS1 Gene. American Journal of Human Genetics.

[18] Field LL (2005). OAS1Splice Site Polymorphism Controlling Antiviral Enzyme Activity Influences Susceptibility to Type 1 Diabetes. Diabetes.

[19] Tessier MC (2006). Type 1 diabetes and the OAS gene cluster: association with splicing polymorphism or haplotype?. Journal of Medical Genetics.

[20] Qu HQ, Polychronakos C (2009). The Type, I. D. G. C. Reassessment of the type I diabetes association of the OAS1 locus. Genes and immunity.

[21] Fedetz M (2006). OAS1 gene haplotype confers susceptibility to multiple sclerosis. Tissue Antigens.

[22] Cagliani R (2012). Identification of a new susceptibility variant for multiple sclerosis in OAS1 by population genetics analysis. Human Genetics.

[23] Gill Tejpal, Asquith Mark, Brooks Stephen R., Rosenbaum James T., Colbert Robert A. (2018). Effects of HLA-B27 on Gut Microbiota in Experimental Spondyloarthritis Implicate an Ecological Model of Dysbiosis. Arthritis & Rheumatology.

[24] Li H (2017). Identification of a Sjogren’s syndrome susceptibility locus at OAS1 that influences isoform switching, protein expression, and responsiveness to type I interferons. PLoS Genet.

[25] Wood H (2010). Multiple sclerosis: OAS1 genotype linked to multiple sclerosis severity. Nat Rev Neurol.

[26] Wang S, Wiley GB, Kelly JA, Gaffney PM (2015). Disease Mechanisms inRheumatology—Tools and Pathways: Defining Functional Genetic Variants in Autoimmune Diseases. Arthritis & Rheumatology (Hoboken, N.j.).

[27] Wright TL (2006). Introduction to Chronic Hepatitis B Infection. Am J Gastroenterol.

[28] Igoe A, Scofield RH (2013). Autoimmunity and Infection in Sjögren’s Syndrome. Current opinion in rheumatology.

[29] Holdgate N, St.Clair EW (2016). Recent advances in primary Sjogren’s syndrome. F1000Research.

[30] Yeh C-C (2016). Association of Sjögrens Syndrome in Patients with Chronic Hepatitis Virus Infection: A Population-Based Analysis. PLoS ONE.

[31] Marcos M (2009). Chronic hepatitis B virus infection in Sjögren’s syndrome. Prevalence and clinical significance in 603 patients. Autoimmunity Reviews.

[32] Aprosin ZG, Lopatkina SV (1993). TN. The hepatitis B virus as a probable etiological factor in Sjögren’s disease. Ter Arkh.

[33] Ward LD, Kellis M (2012). HaploReg: a resource for exploring chromatin states, conservation, and regulatory motif alterations within sets of genetically linked variants. Nucleic Acids Research.

[34] Ward LD, Kellis M (2016). HaploRegv4: systematic mining of putative causal variants, cell types, regulators and target genes for human complex traits and disease. Nucleic Acids Research.

[35] Adrianto I (2012). Two Independent Functional Risk Haplotypes in TNIP1 are Associated with Systemic Lupus Erythematosus. Arthritis and rheumatism.

[36] Wang S (2012). A functional haplotype of UBE2L3 confers risk for Systemic Lupus Erythematosus. Genes and immunity.

[37] Wang S, Wen F, Wiley GB, Kinter MT, Gaffney PM (2013). An Enhancer Element Harboring Variants Associated with Systemic Lupus Erythematosus Engages the TNFAIP3 Promoter to Influence A20 Expression. PLoS Genetics.

[38] Wang S, Wen F, Tessneer KL, Gaffney PM (2016). TALEN-mediated enhancer knockout influences TNFAIP3 gene expression and mimics a molecular phenotype associated with systemic lupus erythematosus. Genes and immunity.

[39] Khattab MA, Eslam M (2012). The Impact of Host Factors on Management of Hepatitis CVirus. Hepatitis Monthly.

[40] Bengsch B, Thimme R, Blum HE (2009). Role of Host Genetic Factors in the Outcome of Hepatitis C VirusInfection. Viruses.

[41] Lim JK (2009). Genetic Variation in OAS1 Is a Risk Factor for Initial Infection with West Nile Virus in Man. PLoS Pathogens.

[42] Lu X (2015). IFN-CSP Inhibiting Hepatitis B Virus in HepG2.2.15 Cells Involves JAK-STAT Signal Pathway. BioMed Research International.

[43] Haddad J (1992). Lymphocytic sialadenitis of Sjögren’s syndrome associated with chronic hepatitis C virus liver disease. The Lancet.

[44] Toussirot E, Lohse A, Wendling D, Mougin C (2000). Sjögren’s syndrome occurring after hepatitis B vaccination. Arthritis & Rheumatism.

[45] Hung T (2015). The Ro60 autoantigen binds endogenous retroelements and regulates inflammatory gene expression. Science.

[46] Weller ML (2016). Hepatitis Delta Virus Detected in Salivary Glands of Sjogren’s Syndrome Patients and Recapitulates a Sjogren’s Syndrome-Like Phenotype *in Vivo*. Pathog Immun.

