# Publisher Correction: A functional variant in the OAS1 gene is associated with Sjögren’s syndrome complicated with HBV infection

**DOI:** 10.1038/s41598-018-35438-9

**Published:** 2018-11-14

**Authors:** Xianjun Liu, Hongcun Xing, Wenjing Gao, Di Yu, Yuming Zhao, Xiaoju Shi, Kun Zhang, Pingya Li, Jiaao Yu, Wei Xu, Hongli Shan, Kaiyu Zhang, Wanguo Bao, Xueqi Fu, Sirui Yang, Shaofeng Wang

**Affiliations:** 1 The Bethune Institute of Epigenetic Medicine, The First Hospital of Jilin University, Changchun, China; 2 College of Life Sciences, The University of Jilin, Changchun, China; 3 Department of Hepatobiliary and Pancreatic Surgery, The First Hospital of Jilin University, Changchun, China; 4 The Research Center, The Second Hospital of Jilin University, Changchun, China; 5 The College of Pharmacy, The University of Jilin, Changchun, China; 6 Department of Burn Surgery, The First Hospital of Jilin University, Changchun, China; 7 Department of Clinical Laboratory, The First Hospital of Jilin University, Changchun, China; 8 Department of infectious Diseases, The First Hospital of Jilin University, Changchun, China; 9 Center of Pediatrics, Institute of Pediatrics, The First Hospital of Jilin University, Changchun, China

Correction to: *Scientific Reports* 10.1038/s41598-017-17931-9, published online 14 December 2017

The original version of this Article contained a typographical error in the spelling of the author Shaofeng Wang, which was incorrectly given as Shafeng Wang. This has now been corrected in the PDF and HTML versions of the Article.

