# Supplementary material for: A functional variant in the OAS1 gene is associated with Sjögren’s syndrome complicated with HBV infection
Source: Sci Rep. 2017 Dec 14;7:17571. doi: 10.1038/s41598-017-17931-9 (PMC5730593; doi:10.1038/s41598-017-17931-9)
Supplement: Supplementary file 1 — Supplementary Dataset [file 41598_2017_17931_MOESM1_ESM.docx]

**Title Page**

**A functional variant in the OAS1 gene is associated with Sjögren’s syndrome complicated with HBV infection**

Xianjun Liu^1,10^, Hongcun Xing^2,10^, Wenjing Gao^1,2^, Di Yu^1^, Yuming Zhao^2^, Xiaoju Shi^3^, Kun Zhang^4^, Pingya Li^5^, Jiaao Yu^6^, Wei Xu^7^, Hongli Shan^7^, Kaiyu Zhang^8^, Wanguo Bao^8^, Xueqi Fu^2^, Sirui Yang^9,^*, Shaofeng, Wang^1,9,^*

**Corresponding Author:** Shaofeng Wang, M.D., Ph.D.

The Bethune Institute of Epigenetic Medicine

The First Hospital of Jilin University

519 East Minzhu Ave.

Changchun City, Jilin Province, China 130061

Phone: +86 (431) 881808172

E-mail: shaofengwang@jlu.edu.cn

**Affiliations:**

- 1. The Bethune Institute of Epigenetic Medicine, The First Hospital of Jilin University, China

2. College of Life Sciences, Jilin University, Changchun, China

3. Department of Hepatobiliary and Pancreatic Surgery, The First Hospital of Jilin University, China

4. The Research Center, The Second Hospital of Jilin University, China

5. The College of Pharmacy, Jilin University, China

6. Department of Burn Surgery, The First Hospital of Jilin University, China

7. Department of Clinical Laboratory, The First Hospital of Jilin University, China

8. Department of infectious Diseases, The First Hospital of Jilin University, China

9. Center of Pediatrics, Institute of Pediatrics, The First Hospital of Jilin University, China

10. These two authors contributed equally to this work

*. Corresponding authors

**This file includes supplementary tables and figures.**

**Supplementary Table 1**

Demographics of the 588 SS cases and 1455 independent controls

**Supplementary Table 2**

SNPs in LD (r^2^ > 0.95) with rs10774671 in an Asian population

**Supplementary Table 3**

Primers used in the current study

**Supplementary Figure 1**

Sanger sequencing of randomly selected individuals confirms the genotypes at the rs10774671 variant.

**Supplementary Figure 2**

Relative locations of SNPs in LD with rs10774671 to OAS1 and OAS3 genes

**Supplementary Figure 3**

Gene expression analysis of OAS3 in PBMCs from SS cases and healthy controls

**Supplementary Figure 4**

Gene expression analysis of OAS3 in EBV-transformed B cells from patients with SS in resting and stimulated conditions.

**Supplementary Figure 5**

Function of rs10774671 in regulating mRNA expression of isoforms of OAS1 in PBMC from patients with SS.

**Full-length Gels and Blots (Original Figures)**

**Supplementary Tables:**

**Supplementary Table 1**

**Demographics of the 588 cases and 1455 independent controls**

|  |  | **Control** | **SS** | ***P*** |
| --- | --- | --- | --- | --- |
| **Age** | | 49.57 ± 18.36 | 50.15 ± 17.13 | 0.268 |
|  | > 30 | 1281 | 519 | 0.514 |
|  | < 30 | 173 | 69 |  |
| **Gender** | |  |  |  |
|  | Male | 141 | 61 | 0.383 |
|  | Female | 1314 | 527 |  |
| **Total** | | **1455** | **588** |  |

**Supplementary Table 2**

**SNPs in LD (r^2^ > 0.95) with rs10774671 in an Asian population**

| **Chr** | **Pos** | **r^2^** | **D'** | **Variant** | **Ref** | **Alt** | **Motifs changed** | **Genes** |
| --- | --- | --- | --- | --- | --- | --- | --- | --- |
| 12 | 112912991 | 1 | 1 | rs2057778 | G | T | Foxa, Hbp1 | *OAS1* |
| 12 | 112913715 | 1 | 1 | rs2285934 | T | G | NF-kappaB, STAT, TCF4 | *OAS1* |
| **12** | **112919388** | **1** | **1** | **rs10774671** | **G** | **A** | **TCF4** | ***OAS1*** |
| 12 | 112919404 | 1 | 1 | rs1131476 | G | A,C,T |  | *OAS1* |
| 12 | 112919432 | 1 | 1 | rs1051042 | G | C | Nanog, SETDB1, SIX5 | *OAS1* |
| 12 | 112919637 | 1 | 1 | rs2660 | G | A | HNF4, RXR::LXR | *OAS1* |
| 12 | 112920301 | 1 | 1 | rs7135577 | A | G | 5 altered motifs | *OAS1* |
| 12 | 112920986 | 1 | 1 | rs4767024 | T | C | Gfi1, Gfi1b | *OAS1* |
| 12 | 112920989 | 1 | 1 | rs4767025 | C | T |  | *OAS1* |
| 12 | 112921327 | 1 | 1 | rs4767026 | A | G | Nkx6-1, Zfp410 | *OAS1* |
| 12 | 112921352 | 1 | 1 | rs4767027 | T | C | 4 altered motifs | *OAS1* |
| 12 | 112921383 | 1 | 1 | rs4767028 | A | G | DMRT2 | *OAS1* |
| 12 | 112921513 | 1 | 1 | rs4767029 | G | A |  | *OAS1* |
| 12 | 112921772 | 1 | 1 | rs4767030 | C | T | HDAC2 | *OAS1* |
| 12 | 112921898 | 1 | 1 | rs10850092 | C | G |  | *OAS1* |
| 12 | 112922220 | 0.99 | 1 | rs6489864 | A | G | 4 altered motifs | *OAS1* |
| 12 | 112922497 | 0.99 | 1 | rs6489865 | A | G | CDP | *OAS1* |
| 12 | 112922663 | 0.99 | 1 | rs10850093 | C | T | AP-2, RXRA, SF1 | *OAS1* |
| 12 | 112922758 | 0.99 | 1 | rs10850094 | T | C | 6 altered motifs | *OAS1* |
| 12 | 112922770 | 0.99 | 1 | rs10850095 | T | C |  | *OAS1* |
| 12 | 112922932 | 0.99 | 1 | rs10774672 | G | T | BRCA1 | *OAS1* |
| 12 | 112923312 | 0.99 | 1 | rs10850097 | C | T |  | *OAS1* |
| 12 | 112923353 | 0.99 | 1 | rs10774673 | C | T | Spz1 | *OAS1* |
| 12 | 112923369 | 0.99 | 1 | rs10774674 | T | C | DMRT7, GATA, Sox | *OAS1* |
| 12 | 112923638 | 0.99 | 1 | rs11066451 | G | A | Foxd3, Foxp1, Pax-4 | *OAS1* |
| 12 | 112923769 | 0.99 | 1 | rs11066452 | G | T | 6 altered motifs | *OAS1* |
| 12 | 112924253 | 0.99 | 1 | rs10850098 | G | C | 6 altered motifs | *OAS1* |
| 12 | 112924602 | 0.99 | 1 | rs10774676 | A | G | IRC900814 | *OAS1* |
| 12 | 112924616 | 0.99 | 1 | rs10774677 | G | A | 4 altered motifs | *OAS1* |
| 12 | 112924946 | 0.99 | 1 | rs4767031 | C | G |  | *OAS1* |
| 12 | 112925169 | 0.99 | 1 | rs4766663 | A | G | Nr2f2, Pax-5, RXRA | *OAS1* |
| 12 | 112925192 | 0.99 | 1 | rs4766664 | T | G | 5 altered motifs | *OAS1* |
| 12 | 112925272 | 0.99 | 1 | rs4767032 | T | G | 4 altered motifs | *OAS1* |
| 12 | 112925373 | 0.99 | 1 | rs4988618 | G | A | 6 altered motifs | *OAS1* |
| 12 | 112925603 | 0.99 | 1 | rs6489866 | A | G | SIX5, Znf143 | *OAS1* |
| 12 | 112925745 | 0.99 | 1 | rs6489867 | C | T | CDP, NRSF | *OAS1* |
| 12 | 112925887 | 0.99 | 1 | rs6489868 | G | C | Hoxa9 | *OAS1* |
| 12 | 112926117 | 0.99 | 1 | rs6489869 | A | C | 9 altered motifs | *OAS1* |
| 12 | 112926167 | 0.99 | 1 | rs6489870 | G | A | AP-2, Glis2, Spz1 | *OAS1* |
| 12 | 112926666 | 0.99 | 1 | rs4767034 | A | G | Esr2, SIX5 | *OAS1* |
| 12 | 112926699 | 0.99 | 1 | rs10850099 | A | G | Irf | *OAS1* |
| 12 | 112926707 | 0.99 | 1 | rs10850100 | T | C | Gm397, Nrf-2 | *OAS1* |
| 12 | 112926828 | 0.99 | 1 | rs4766667 | G | A | Pax-4, p53 | *OAS1* |
| 12 | 112927068 | 0.99 | 1 | rs4766670 | A | G | GR, Hoxa5, NRSF | *OAS1* |
| 12 | 112927260 | 0.99 | 1 | rs4766671 | A | G | 5 altered motifs | *OAS1* |
| 12 | 112927356 | 0.99 | 1 | rs4766673 | A | G |  | *OAS1* |
| 12 | 112927396 | 0.99 | 1 | rs4766674 | G | T | E4F1, Hbp1, Pax-3 | *OAS1* |
| 12 | 112927418 | 0.99 | 1 | rs10716630 | CG | C | MAZ, Pou1f1 | *OAS1* |
| 12 | 112927648 | 0.99 | 1 | rs4766675 | A | T | 4 altered motifs | *OAS1* |
| 12 | 112927776 | 0.99 | 1 | rs4766676 | C | T | HDAC2, Sin3Ak-20 | *OAS1* |
| 12 | 112927862 | 0.99 | 1 | rs7304898 | T | C | Mef2, STAT | *OAS1* |
| 12 | 112927882 | 0.99 | 1 | rs7315441 | G | T | Evi-1 | *OAS1* |
| 12 | 112927998 | 0.99 | 1 | rs7305035 | T | C | 5 altered motifs | *OAS1* |
| 12 | 112928023 | 0.99 | 1 | rs7316586 | C | T | Evi-1, Maf, PEBP | *OAS1* |
| 12 | 112928244 | 0.99 | 1 | rs916972 | G | T | HNF4, RXRA | *OAS1* |
| 12 | 112928886 | 0.99 | 1 | rs7134391 | G | A | TLX1, NFIC | *OAS1* |
| 12 | 112929094 | 0.99 | 1 | rs7306205 | A | G | 4 altered motifs | *OAS1* |
| 12 | 112929504 | 0.99 | 1 | rs1859336 | C | T | Hand1, Mef2 | *OAS1* |
| 12 | 112929520 | 0.99 | 1 | rs1859335 | C | T | RORalpha1 | *OAS1* |
| 12 | 112929527 | 0.99 | 1 | rs1859334 | C | T | AP-2 | *OAS1* |
| 12 | 112929538 | 0.99 | 1 | rs2384071 | A | G | 4 altered motifs | *OAS1* |
| 12 | 112929617 | 0.99 | 1 | rs2384072 | T | C | 6 altered motifs | *OAS1* |
| 12 | 112929790 | 0.99 | 1 | rs2384073 | A | G | 7 altered motifs | *OAS1* |
| 12 | 112930090 | 0.99 | 1 | rs6489874 | A | G |  | *OAS1* |
| 12 | 112930250 | 0.99 | 1 | rs6489876 | G | A | 12 altered motifs | *OAS1* |
| 12 | 112930274 | 0.99 | 1 | rs6489877 | A | G | NRSF | *OAS1* |
| 12 | 112930320 | 0.99 | 1 | rs6489878 | A | G | Ik-2, Zfp410 | *OAS1* |
| 12 | 112930444 | 0.99 | 1 | rs7298184 | C | A | HNF4, Sox | *OAS1* |
| 12 | 112930700 | 0.99 | 1 | rs12322160 | A | G | LBP-1 | *OAS1* |
| 12 | 112930800 | 0.99 | 1 | rs7132404 | T | C | E2F, GR | *OAS1* |
| 12 | 112931372 | 0.99 | 1 | rs4767036 | T | G | HDAC2, HMG-IY, Pax-6 | *OAS1* |
| 12 | 112931954 | 0.99 | 1 | rs7967461 | G | C | KAP1, Myf, Pou6f1 | *OAS1* |
| 12 | 112932622 | 0.99 | 1 | rs1154970 | A | C | 4 altered motifs | *OAS1 - OAS3* |
| 12 | 112933309 | 0.99 | 1 | rs1859332 | T | C | Egr-1, Irf, Pax-5 | *OAS1 - OAS3* |
| 12 | 112933841 | 0.99 | 1 | rs9971885 | A | C | Zbtb3 | *OAS1 - OAS3* |
| 12 | 112934168 | 0.99 | 1 | rs57484342 | A | G | TCF12 | *OAS1 - OAS3* |
| 12 | 112934172 | 0.99 | 1 | rs61266553 | A | G | TCF12 | *OAS1 - OAS3* |
| 12 | 112934734 | 0.99 | 1 | rs7966314 | A | G | Mef2, TATA | *OAS1 - OAS3* |
| 12 | 112934999 | 0.99 | 1 | rs4767037 | A | C | Pou2f2 | *OAS1 - OAS3* |
| 12 | 112935061 | 0.99 | 1 | rs1981557 | G | C |  | *OAS1 - OAS3* |
| 12 | 112935156 | 0.99 | 1 | rs1981556 | C | G | PPAR | *OAS1 - OAS3* |
| 12 | 112935172 | 0.99 | 1 | rs1981555 | G | A | HNF4, NR4A, SREBP | *OAS1 - OAS3* |
| 12 | 112935758 | 0.99 | 1 | rs3759376 | A | G | 21 altered motifs | *OAS1 - OAS3* |
| 12 | 112935783 | 0.99 | 1 | rs3759375 | A | G |  | *OAS1 - OAS3* |
| 12 | 112936212 | 0.99 | 1 | rs4767040 | G | C | Mef2 | *OAS1 - OAS3* |
| 12 | 112936943 | 0.99 | 1 | rs10774679 | C | T | BCL, PPAR, Pax-5 | *OAS1 - OAS3* |
| 12 | 112937231 | 0.99 | 1 | rs7132797 | A | C | CAC-binding-protein, SP1, STAT | *OAS1 - OAS3* |
| 12 | 112938178 | 0.99 | 1 | rs1156361 | T | C | GR, Zfp187 | *OAS1 - OAS3* |
| 12 | 112938526 | 0.99 | 1 | rs1859331 | C | A,G,T |  | *OAS3* |
| 12 | 112939108 | 0.99 | 1 | rs7299132 | T | A |  | *OAS3* |
| 12 | 112940017 | 0.99 | 1 | rs6489879 | G | A | TCF12, p53 | *OAS3* |
| 12 | 112940276 | 0.99 | 1 | rs4238033 | T | A | 7 altered motifs | *OAS3* |
| 12 | 112941234 | 0.99 | 1 | rs7955267 | C | T |  | *OAS3* |
| 12 | 112942203 | 0.99 | 1 | rs10735079 | G | A | AP-2 | *OAS3* |
| 12 | 112942466 | 0.99 | 1 | rs6489880 | C | T | Ik-1 | *OAS3* |
| 12 | 112942724 | 0.99 | 1 | rs7980275 | T | A | 8 altered motifs | *OAS3* |
| 12 | 112942903 | 0.99 | 1 | rs7977345 | A | T |  | *OAS3* |
| 12 | 112943412 | 0.99 | 1 | rs6489881 | A | T | RREB-1 | *OAS3* |

**Supplementary Table 3**

**Primers used in the current study**

| **Primer** | **Sequence** |
| --- | --- |
| OAS1 isoform construct forward | 5’-CCGGAATTCATGATGGATCTCAGAAATACCCCAGCC-3’ |
| OAS1 isoform p46 construct reverse | 5‘-CCGCTCGAGTCAGAGGATGGTGCAGGTCCAGT-3’ |
| OAS1 isoform p48 construct reverse | 5’-CCGCTCGAGTCAGGAGACCTGGGTTCTGTC-3’ |
| OAS1 isoform p42 construct reverse | 5’-CCGCTCGAGCTAATTATTGGTATAGTTCCTTCTGCC-3’ |
| OAS1 isoform p44 construct reverse | 5‘-CCGCTCGAGTCAAGCTTCATGGAGAGGGGCA-3’ |
| OAS1 isoform identify forward | 5’-CTGGCTGAATTACCCATGCTTTA-3’ |
| OAS1 isoform p46/p48 identify reverse | 5’-AACTGGTCCAGATAACACTGGA-3’ |
| OAS1 isoform p42 identify reverse | 5'-GGGAGGGAGTTCACATGTATTCATAT-3' |
| OAS1 isoform p44 identify reverse | 5'-TCTTAGGGGAATGAATTCTGGACAA-3' |
| Total GAPDH forward | 5'-ATGGGGAAGGTGAAGGTCGGA-3' |
| Total GAPDH reverse | 5'-TTACTCCTTGGAGGCCATGTGG-3' |
| Total OAS1 qPCR forward | 5’-GAAGGAAAGGTGCTTCCGAGGTAG-3’ |
| Total OAS1 qPCR reverse | 5’-AAGACAACCAGGTCAGCGTCAGAT-3’ |
| OAS1 isoform p46 qPCR forward | 5’-CTGGCTGAATTACCCATGCTTTA-3’ |
| OAS1 isoform p46 qPCR reverse | 5’-TCTGATACCTCCTGGGATCGT-3’ |
| OAS1 isoform p48 qPCR forward | 5’-ATTCTGCTGACCCAGCACA-3’ |
| OAS1 isoform p48 qPCR reverse | 5’-AACTGGTCCAGATAACACTGGA-3’ |
| Total OAS2 qPCR forward | 5’-AACACCATCTGTGACGTCCTGCA-3’ |
| Total OAS2 qPCR reverse | 5’-AAGGGTACCATCGGAGTTGCCTC-3’ |
| Total OAS3 qPCR forward | 5’-CGCGGGTGCTGAAAACTGTCAA-3’ |
| Total OAS3 qPCR reverse | 5’-TCTGGTCCACATAGCTCTTGAAGCA-3’ |
| GAPDH qPCR forward | 5’-CCATGAGAAGTATGACAACAGC-3’ |
| GAPDH qPCR reverse | 5’-TGGTCATGAGTCCTTCCAC-3’ |
| HBV DNA forward* | 5'-CACATCAGGATTCCTAGGACC-3' |
| HBV DNA reverse* | 5'-GGTGAGTGATTGGAGGGTTG-3' |
| rs10774671 TaqMan primer forward | 5’-AAGACTCCCTGATGTGATCATGTG-3’ |
| rs10774671 TaqMan primer reverse | 5’-TCATCGTCTGCACTGTTGCTT-3’ |
| rs10774671 TaqMan probe ‘A’ | 5’-CTCACCCTTTCAAGC-3’ |
| rs10774671 TaqMan probe ‘G’ | 5’-TCACCCTTTCAGGCTG-3’ |

**Supplementary Figures**

**Supplementary Figure 1**

**Sanger sequencing of randomly selected individuals confirms the genotypes at the rs10774671 variant.**

**Supplementary Figure 2**

**Relative location of SNPs in LD with rs10774671 to *OAS1* and *OAS3* genes.** SNPs in LD (r^2^ is greater than 0.99) with rs10774671 have been identified using Haploreg 4.1 and were listed in the figure using the table browser from the UCSC genome browser.

**Supplementary Figure 3**

**Gene expression analysis of *OAS3* in PBMCs from patients with SS and healthy controls.** Messenger RNA expression levels of *OAS3* in PBMCs from patients with SS and controls were determined by RT-qPCR and were normalized to *GAPDH* controls. Comparisons were made between groups stratified by genotypes at rs10774671. Statistically differences were calculated by one-way ANOVA. No statistically significant differences were found across groups.

**Supplementary Figure 4**

**Gene expression analysis of *OAS3* in EBV-transformed B cells from patients with SS in resting and stimulated conditions.** Twelve EBV-transformed B cell lines were generated using PBMCs from patients with SS and were stimulated with TNF alpha for 2 hours. Gene expression of *OAS3* was determined by RT-qPCR assays and was normalized to a GAPDH control. Statistical differences were calculated using one-way ANOVA. We did not observe significant differences between groups in either resting or stimulated conditions.

**Supplementary Figure 5.**

**Function of rs10774671 in regulating mRNA expression of isoforms of OAS1 in PBMC from patients with SS.** The risk allele A of rs10774671 decreases expression of OAS1 TV1 (A) and increases in mRNA expression of OAS1 TV2 (B), TV3 (C), ant TV4 (D). Each data point represents a SS patient. Statistically differences were calculated by one-way ANOVA.

**Full-length Gels and Blots (Original Figures)**

**Original Figure 1. Expression of isoforms OAS1_V1 and OAS1_V3 in PBMC**

Lines from the left to the right: Marker, Individual 1 with GG genotype, Individual 2 with GG genotype, Individual 3 with GA genotype, Individual 4 with GA genotype, Individual 5 with AA genotype, and Individual 6 with AA genotype.

**Original Figure 2. Expression of isoform OAS1_V2 in PBMC**

Lines from the left to the right: Marker, a negative control, Individual 1 with GG genotype, Individual 2 with GG genotype, Individual 3 with GA genotype, Individual 4 with GA genotype, Individual 5 with AA genotype, and Individual 6 with AA genotype.

**Original Figure 3. Expression of isoform OAS1_V4 in PBMC**

Lines from the left to the right: Marker, Individual 1 with GG genotype, Individual 2 with GG genotype, Individual 3 with GA genotype, Individual 4 with GA genotype, Individual 5 with AA genotype, and Individual 6 with AA genotype.

**Original Figure 4. Loading control GAPDH in PBMC**

**
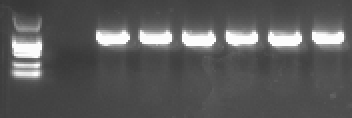
**

Lines from the left to the right: Marker, Individual 1 with GG genotype, Individual 2 with GG genotype, Individual 3 with GA genotype, Individual 4 with GA genotype, Individual 5 with AA genotype, and Individual 6 with AA genotype.

**Original Figure 5. Expression of isoforms OAS1_V1 and OAS1_V3 in EBV cells**

Lines from the left to the right: Marker, Individual 1 with GG genotype, Individual 2 with GG genotype, Individual 3 with GA genotype, Individual 4 with GA genotype, Individual 5 with AA genotype, and Individual 6 with AA genotype.

**Original Figure 6. Expression of isoform OAS1_V2 in EBV cells**

Lines from the left to the right: Marker, a negative control, Individual 1 with GG

genotype, Individual 2 with GG genotype, Individual 3 with GA genotype, Individual 4

with GA genotype, Individual 5 with AA genotype, and Individual 6 with AA genotype.

**Original Figure 7. Expression of isoform OAS1_V4 in EBV cells**

Lines from the left to the right: Marker, Individual 1 with GG genotype, Individual 2 with GG genotype, Individual 3 with GA genotype, Individual 4 with GA genotype, Individual 5 with AA genotype, and Individual 6 with AA genotype.

**Original Figure 8. Loading control GAPDH in EBV cells**

Lines from the left to the right: Marker, Individual 1 with GG genotype, Individual 2 with GG genotype, Individual 3 with GA genotype, Individual 4 with GA genotype, Individual 5 with AA genotype, and Individual 6 with AA genotype.

**Original Figure 9. Overexpression of various isoforms of OAS1 in HepG2 cells**

The OAS1 proteins expression were measured by chemiluminescent western blotting with antibody against Xpress tag. Lines from the left to the right: empty vector control, OAS1 isoform 1, OAS1 isoform 2, OAS1 isoform 3, and OAS1 isoform 4.

**Original Figure 9. Expression of the hepatitis B core protein in HepG2 cells**

The expression of hepatitis B core protein was detected by using chemiluminescent western blotting with anti-HBc antibody. Lines from the left to the right: empty vector control, OAS1 isoform 1, OAS1 isoform 2, OAS1 isoform 3, and OAS1 isoform 4.

**Original Figure 10. Detection of beta-actin in the HepG2 cells.**

The expressions of beta-actin controls in each sample were measured by using chemiluminescent western blotting with anti-beta-actin antibody**.** Lines from the left to the right: empty vector control, OAS1 isoform 1, OAS1 isoform 2, OAS1 isoform 3, and OAS1 isoform 4.
